# Activity of D-amino acid oxidase is widespread in the human central nervous system

**DOI:** 10.3389/fnsyn.2014.00014

**Published:** 2014-06-10

**Authors:** Jumpei Sasabe, Masataka Suzuki, Nobuaki Imanishi, Sadakazu Aiso

**Affiliations:** Department of Anatomy, Keio University School of MedicineShinjuku-ku, Tokyo, Japan

**Keywords:** D-serine, D-amino acid oxidase, enzyme histochemistry, human brain, white matter, motor systems, amyotrophic lateral sclerosis, schizophrenia

## Abstract

It has been proposed that D-amino acid oxidase (DAO) plays an essential role in degrading D-serine, an endogenous coagonist of *N*-methyl-D-aspartate (NMDA) glutamate receptors. DAO shows genetic association with amyotrophic lateral sclerosis (ALS) and schizophrenia, in whose pathophysiology aberrant metabolism of D-serine is implicated. Although the pathology of both essentially involves the forebrain, in rodents, enzymatic activity of DAO is hindbrain-shifted and absent in the region. Here, we show activity-based distribution of DAO in the central nervous system (CNS) of humans compared with that of mice. DAO activity in humans was generally higher than that in mice. In the human forebrain, DAO activity was distributed in the subcortical white matter and the posterior limb of internal capsule, while it was almost undetectable in those areas in mice. In the lower brain centers, DAO activity was detected in the gray and white matters in a coordinated fashion in both humans and mice. In humans, DAO activity was prominent along the corticospinal tract, rubrospinal tract, nigrostriatal system, ponto-/olivo-cerebellar fibers, and in the anterolateral system. In contrast, in mice, the reticulospinal tract and ponto-/olivo-cerebellar fibers were the major pathways showing strong DAO activity. In the human corticospinal tract, activity-based staining of DAO did not merge with a motoneuronal marker, but colocalized mostly with excitatory amino acid transporter 2 and in part with GFAP, suggesting that DAO activity-positive cells are astrocytes seen mainly in the motor pathway. These findings establish the distribution of DAO activity in cerebral white matter and the motor system in humans, providing evidence to support the involvement of DAO in schizophrenia and ALS. Our results raise further questions about the regulation of D-serine in DAO-rich regions as well as the physiological/pathological roles of DAO in white matter astrocytes.

## Introduction

D-Amino acid oxidase (DAO) is an FAD-dependent enzyme that catalyzes the oxidative deamination of D-isomers of neutral and polar amino acids. Among substrates of mammalian DAO, D-serine is by far the most abundant in the mammalian brain and is therefore regarded as its major substrate. In fact, the D-serine level is inversely related to DAO activity in rodent brain (Morikawa et al., 2001; Wang and Zhu, 2003; Miyoshi et al., 2009). Since D-serine is an endogenous coagonist of *N-methyl-D-aspartate* (NMDA) glutamate receptors located at the synapse (Mothet et al., 2000; Panatier et al., 2006; Basu et al., 2009; Papouin et al., 2012), and also serves as an endogenous ligand at the δ2 glutamate receptor (Kakegawa et al., 2011), DAO has the potential to modulate neurotransmission through D-serine.

D-Serine is converted from L-serine by a PLP-dependent enzyme, serine racemase (SR) (Wolosker et al., 1999), which is highly expressed in the brains of mammals including humans (Verrall et al., 2007; Balu et al., 2014). Due to forebrain-shifted expression of SR (Miya et al., 2008), D-serine exists in the cerebral cortex at approximately 100–150 nmol/g tissue in humans (Kumashiro et al., 1995; Hamase et al., 2005; Bendikov et al., 2007), and at 300–350 nmol/g tissue in rodents (Hashimoto et al., 1993; Nagata et al., 1994; Morikawa et al., 2001) in most studies. In contrast, abundant DAO in the hindbrain and spinal cord is believed to keep D-serine at a low level in the regions; the cerebellum of humans (Kumashiro et al., 1995) and rodents (Hashimoto et al., 1993; Nagata et al., 1994; Morikawa et al., 2001; Wang and Zhu, 2003) contains D-serine at less than 30 nmol/g tissue, although the results vary in each study depending on the sensitivity of the method used to determine small amounts of D-serine.

Based upon activity assays, rodent DAO has been viewed as an enzyme expressed in the hindbrain and spinal cord (Neims et al., 1966; Arnold et al., 1979; Horiike et al., 1994). Its activity has also been detected in the forebrain in some studies, albeit at only a small fraction of that seen in cerebellum (Neims et al., 1966; Weimar and Neims, 1977; Madeira et al., 2008). This view is consistent with the evidence that D-serine is unchanged or only minimally increased in the cerebral cortex, in contrast to its marked increase in the cerebellum in mice lacking DAO activity due to a mutation of G181R (Hashimoto et al., 1993; Morikawa et al., 2001; Miyoshi et al., 2009). On the other hand, the distribution of human DAO based on its activity remains unknown. In contrast to rodents, human DAO may also exist, to some extent, in the forebrain region. Several lines of evidence support this possibility. First, DAO immunoreactivity has been detected in the homogenates of human cerebral cortex (Bendikov et al., 2007; Sacchi et al., 2008); second, the human cerebral cortex contains only one-third of the D-serine level found in the corresponding part of the mouse brain. Most importantly, DAO shows genetic associations with human neurological diseases such as schizophrenia (Chumakov et al., 2002; Schumacher et al., 2004) and amyotrophic lateral sclerosis (ALS) (Mitchell et al., 2010), both of which essentially involve forebrain pathology.

ALS is an adult-onset neurodegenerative disorder characterized by selective loss of upper and lower motor neurons. Glutamate excitotoxicity is implicated in its pathogenesis (Bruijn et al., 2004; Van Den Bosch et al., 2006), and the accumulation of D-serine due to reduced activity of DAO possibly exacerbates hyperactivity of motor neurons via NMDA receptors (Sasabe et al., 2007, 2012; Paul et al., 2014). On the other hand, schizophrenia is a severely debilitating psychiatric condition, characterized by psychotic features, negative symptoms, and cognitive defects. A model featuring glutamatergic hypofunction with accompanying disinhibition of the dopaminergic system has gained significant support (Carlsson and Carlsson, 1990; Lisman et al., 2008), and reduced D-serine due to activation of DAO triggered hypofunction of NMDA receptors may be partly responsible (Kapoor et al., 2006; Burnet et al., 2008; Madeira et al., 2008; Labrie et al., 2012; Van Horn et al., 2013). In the present study, we used post-mortem brains with intact DAO enzymatic activity and demonstrated the distribution of DAO using an activity-based staining method, with a major focus on differences between humans and mice in the forebrain region and motor pathways.

## Materials and methods

### Human samples

Postmortem specimens were collected from cadavers donated after death with prior consent at Keio University School of Medicine. A male cadaver who had died of gastric cancer at 89 y/o was examined at 13 h postmortem, a male cadaver who had died of gastrointestinal hemorrhage at 86 y/o was at 28 h, and a female cadaver who had died of bile duct cancer at 92 y/o was at 36 h. All of them were Japanese. Neuropathological assessment of their brain did not identify any metastasis of cancer or neurodegenerative abnormalities other than minor age-related changes. The specimens were dissected after perfusion from the thoracic aorta with ice-cold phosphate buffer-saline (PBS, pH 7.4). The pieces of brain tissue were processed individually for enzyme activity assay or histological analysis.

### Animals

All experiments on animals were carried out in accordance with institutional guidelines. The study protocol was approved by the Animal Experiment Committee of Keio University. C57BL/6J mice were obtained from CLEA Japan (Tokyo, Japan).

### Enzyme activity assay of DAO

The dissected pieces of human tissue for enzyme activity assay were kept at −80°C without fixation until use. C57BL/6J male mice at 8 weeks of age were euthanized and maintained at 4°C. Mouse tissue was collected at 0, 12, 24, or 48 h after death and kept at −80°C until use. Tissue was homogenized in 7 mM sodium pyrophosphate (pH 8.3) at 3500 rpm for 2 min using Micro Smash MS-100 (Tomy, Tokyo, Japan) and centrifuged at 5500 × g for 10 min. The activity of DAO was determined as described previously (Sasabe et al., 2012, 2014). Briefly, 50 μ l tissue lysate was added to a mixture [150 μ l of 100 mM D-Ala, 100 μ l of 0.1 mM flavin adenine dinucleotide (FAD), 150 μ l of 700 units/ml catalase in 133 mM sodium pyrophosphate (pH 8.3), and 50 μ l of 70% v/v MeOH], processed with constant agitation at 37°C for 30–60 min, and terminated by adding 500 μ l of 10% trichloroacetic acid. To 250 μ l of the supernatant solution were added 250 μ l of 5 M KOH and 250 μ l of 0.5% 4-amino-3-hydrazino-5-mercapto-1,2,4-triazole in 0.5 M HCl. After 15 min incubation at room temperature, 250 μ l of 0.75% KIO_4_ in 0.2 M KOH was added to the mixture with vigorous shaking, and absorbance at 550 nm was measured. DAO activity was calculated as described by Watanabe et al. (1978) and expressed as the amount of D-alanine oxidized per minute per milligram of protein.

### Histological analysis

The human tissue pieces for enzyme histochemistry were fixed in 2% paraformaldehyde in PBS on ice for 2 h. Mice were anesthetized with diethyl ether and perfused transcardially with ice-cold PBS and subsequently with 2% paraformaldehyde in PBS. Human and mouse tissues were then cryoprotected in a 20% sucrose solution in PBS at 4°C overnight. They were frozen in Tissue-Tek O.C.T. Compound (Sakura Finetek Japan, Tokyo, Japan). Sections 15 μm thick were sliced on a cryostat at −19°C and stored at −80°C until they were used.

For double staining, the procedure for immunofluorescence was done prior to that of the enzyme histochemistry. Sections were rinsed in PBS and incubated in a blocking solution containing appropriate normal serum (1:60; Vector Laboratories, Cambridgeshire, UK) at room temperature for 1 h. Subsequently, they were incubated in appropriate primary antibodies [non-phosphorylated neurofilament H (npNFH), SMI32 (1:100; Covance, Princeton, NJ, USA); glial fibrillary acidic protein (GFAP) (1:300; Dako, Glostrup, Denmark); and excitatory amino acid transporter 2 (EAAT2/GLT-1), AB1783 (1:100; Merck Millipore, Darmstadt, Germany)] diluted in PBS containing blocking serum at 4°C overnight, rinsed in PBS, and transferred into species-specific secondary antibodies conjugated with Texas-Red (1:250, Jackson ImmunoResearch Laboratories, West Grove, PA, USA) at room temperature for 40 min. The sections were then rinsed again in PBS and incubated in a DAO-activity staining solution [7 mM pyrophosphate buffer (pH 8.3), 0.1% horseradish peroxidase (Sigma-Aldrich, St. Louis, MO, USA), FITC-conjugated tyramide (1:400; Perkin-Elmer, Waltham, MA, USA), 0.065% sodium azide, 0.6% nickel ammonium sulfate, 22 mM D-proline, 20 μM FAD] (Sasabe et al., 2012) at room temperature for 10 min in the dark. They were washed in PBS and mounted using ProLong Gold Antifade Reagent (Invitrogen, Carlsbad, CA, USA).

Sections were imaged using a BZ-9000 fluorescent microscope (Keyence, Osaka, Japan) for joint images and a Zeiss LSM 510 confocal microscope (Carl Zeiss, Oberkochen, Germany) for double-stained images.

All images in the figures are from the 89 y/o male. The results of DAO-enzyme histochemistry in Figure 2 were also confirmed in the sections from the 92 y/o female.

### Statistics

All values in the figures of this study indicate means ± standard error of mean (SEM). Statistical analyses for the experiments were performed with one-way ANOVA followed by Tukey's multiple comparison test, in which *P* < 0.05 was assessed as significant. All analyses were performed using Prism 5 (GraphPad Software, La Jolla, CA, USA). A sample size of 3 in each group has an 80% power to detect a difference between means of 8.73 (estimated *SD* = 3.0) with a significance level of 0.05, which was calculated using StatMate (GraphPad Software).

## Results

### Tissue DAO activity in human and mouse central nervous systems

**Figure 1:**
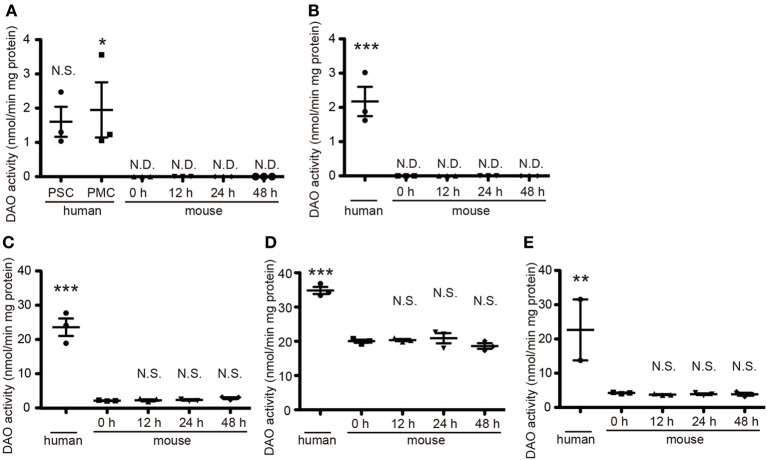
**Quantitative DAO activity in human and mouse tissue**. Tissue DAO activities in human and mouse CNS were measured. Mouse tissues were dissected at indicated hours after the death. **(A)** PSC and PMC of human cerebral cortex (*n* = 3, each), and whole mouse cerebral cortex (*n* = 3, each). **(B)** Posterior limb of human internal capsule (*n* = 3) and mouse striatum (*n* = 3, each). **(C)** Ventral part of human pons (*n* = 3) and mouse brainstem (*n* = 3, each). **(D)** Posterior lobe in hemisphere of human cerebellum (*n* = 3) and whole mouse cerebellum (*n* = 3, each). **(E)** Thoracic parts of human spinal cord (*n* = 2, we could not obtain the spinal cord from 86 y/o male) and of mouse spinal cord (*n* = 3, each). N.D. is “not detected.” N.S. means “not significant.” ^*^P < 0.05, ^**^P < 0.01, ^***^P < 0.001 (One-Way ANOVA followed by Tukey's multiple comparison test, N.S. and asterisks in the figures are comparison to “mouse 0 h”). Data are plotted as the mean ± s.e.m.

DAO activity is restricted to hindbrain and spinal cord in rodents and is strongest in their cerebella (Horiike et al., 1994). To determine DAO activity in human tissue, we obtained a variety of CNS specimens at autopsy gifted from three individuals. In mice, DAO activity did not wane over time until 48 h after death (Figure 1), validating the freshness of the human specimens for DAO activity assay. DAO activity was detected in all the human specimens examined (Figure 1). Surprisingly, the human cerebral cortices of Brodmann area 1–3 (primary somatosensory cortex, PSC) and area 4 (primary motor cortex, PMC) and the internal capsule showed mild DAO activity, while in mice there was no such activity in the cerebral cortex or the striatum, including the internal capsule [Figure 1A, *F*_(5, 12)_ = 6.071, *P* = 0.0050; human PSC, 1.60 ± 0.44; human PMC, 1.95 ± 0.81; mouse 0 h, N.D.; mouse 12 h, N.D.; mouse 24 h, N.D.; mouse 48 h, N.D.] [Figure 1B, *F*_(4, 10)_ = 25.55, *P* < 0.0001; human, 2.17 ± 0.43; mouse 0 h, N.D.; mouse 12 h, N.D.; mouse 24 h, N.D.; mouse 48 h, N.D.] (unit: nmol/min mg protein). Activity in the basilar part of pons and thoracic spinal cord was comparable to that in the cerebellar cortex in humans, whereas it was one-tenth of that in the cerebellum in mice [Figure 1C, *F*_(4, 10)_ = 67.26, *P* < 0.0001; human, 23.62 ± 2.50; mouse 0 h, 2.63 ± 0.09; mouse 12 h, 2.73 ± 0.28; mouse 24 h, 2.84 ± 0.37; mouse 48 h, 2.83 ± 0.27] [Figure 1D, *F*_(4, 10)_ = 53.14, *P* < 0.0001; human, 34.84 ± 1.03; mouse 0 h, 20.04 ± 0.46; mouse 12 h, 20.36 ± 0.32; mouse 24 h, 20.90 ± 1.48; mouse 48 h, 18.63 ± 0.81] [Figure 1E, *F*_(4, 9)_ = 8.467, *P* = 0.0041; human, 22.65 ± 8.89; mouse 0 h, 4.23 ± 0.12; mouse 12 h, 3.76 ± 0.14; mouse 24 h, 3.89 ± 0.23; mouse 48 h, 3.86 ± 0.42] (unit: nmol/min mg protein). These results delineated the difference of DAO distribution in the central nervous system (CNS) between humans and mice.

### Regional activity of DAO in human and mouse brains

Next, we compared the tissue distribution pattern of DAO activity between humans and mice in various regions of brain using a sensitive technique for enzyme histochemistry (Sasabe et al., 2012). This technique is based on covalent binding of fluorescence-conjugated tyramide catalyzed by horseradish peroxidase in the presence of hydrogen peroxide, produced by degradation of externally added D-proline mediated by endogenous DAO. The specificity of this technique to DAO activity was shown in our previous study by using L- instead of D-proline as a substrate of DAO or using sections from mice lacking DAO activity (Sasabe et al., 2012). Its specificity to DAO activity was also tested on human sections; we did not observe any signals when we used L-proline as a substrate (Supplementary Figure 1). We stained sections from two individuals, and staining images from both showed comparable pattern.

#### Cerebral cortex and hippocampus

**Figure 2:**
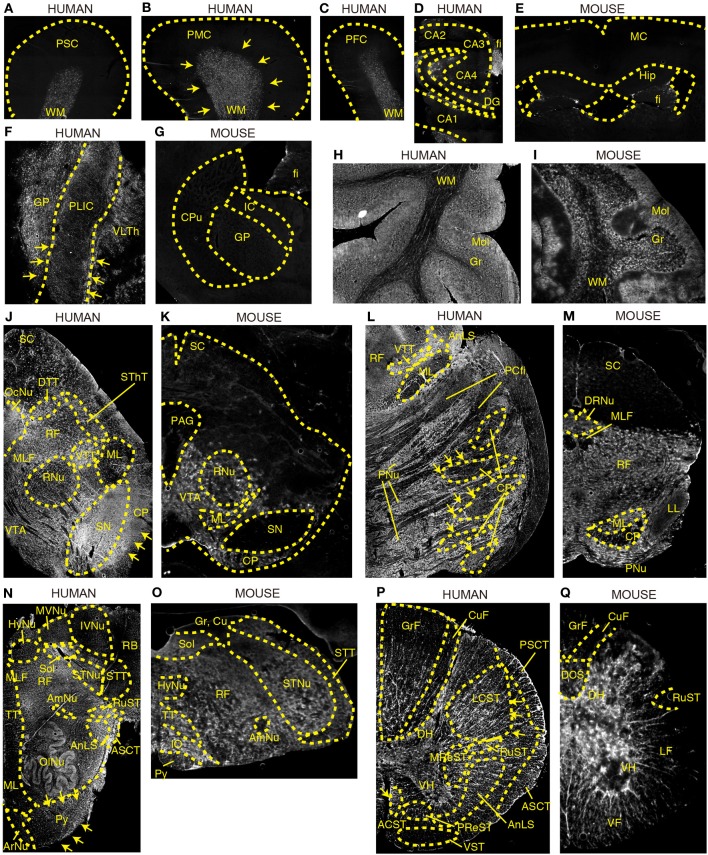
**DAO enzyme histochemistry in human and mouse CNS**. DAO activity was visualized using D-proline as a substrate in various regions of brain. Shown are **(A–C)** human cerebral cortex, **(D)** human hippocampus, **(E)** mouse forebrain including cerebral cortex and hippocampus (a coronal section), **(F)** posterior limb of human internal capsule (a horizontal section), **(G)** mouse striatum and internal capsule (a coronal section), **(H)** posterior lobe in human cerebellar hemisphere, **(I)** mouse cerebellar hemisphere, **(J)** human mesencephalon, **(K)** mouse mesencephalon, **(L)** basilar part of human pons, **(M)** mouse pons, **(N)** human medulla oblongata, **(O)** mouse medulla oblongata, **(P)** human thoracic spinal cord, and **(Q)** mouse thoracic spinal cord. **(J–Q)** Upper sides of the images are dorsal; bottom sides are ventral. **(B,F,J,L,N,P)** Arrows indicate the corticospinal tract. Each image of humans was low magnified to compare them to the corresponding image of mice, and the magnification percentage is different in each image.

DAO activity was found predominantly in the subcortical white matter of Brodmann areas 1–3 (PSC), area 4 (PMC), and area 9 (dorsolateral prefrontal cortex, DPFC) in humans (Figures 2A–C). In the human hippocampus, enzyme reactivity was absent in the pyramidal cell layer of CA1–4 and in the granule cell layer of dentate gyrus (DG), whereas it was slightly detected in the molecular layer and clearly in the fimbria, a band of white matter (Figure 2D). On the other hand, in mice, DAO activity was not observed in either white or gray matter of the cerebral cortex, or in the hippocampus, except slightly in the fimbria (fi) of hippocampus (Hip) (Figure 2E).

#### Internal capsule

The internal capsule consists of three parts: the anterior limb, the posterior limb, and the genu. Within the posterior limb are descending fibers that include corticospinal, corticorubral, and corticoreticular tracts as well as ascending sensory fibers from the thalamus to the cerebral cortex. DAO enzyme activity was visible in the posterior limb of internal capsule (PLIC) as well as in the globus pallidus (GP) and ventral lateral part of thalamus (VLTh) in the human brain (Figure 2F), whereas it was undetectable in the corresponding regions in mice (Figure 2G).

#### Cerebellum

DAO activity was localized predominantly to the cortex in the cerebellar hemisphere of both humans and mice (Figures 2H,I). The activity was strongest in the molecular layer (Mol), patchy in the granular layer (Gr), and mild in the white matter (WM).

#### Mesencephalon

DAO activity was seen throughout the mesencephalon in humans (Figure 2J), while it was restricted to the ventral side in mice (Figure 2K). In humans, DAO activity was strong in substantia nigra (SN), cerebral peduncle (CP), and oculomotor nucleus (OcuNu); moderate in ventral and dorsal trigeminal tracts (VTT and DTT), spinothalamic tract (SThT), and medial longitudinal fasciculus (MLF); mild in ventral tegmental area (VTA) and reticular formation (RF); and slight in red nucleus (RNu), medial lemniscus (ML), and superior colliculus (SC) (Figure 2J). In mice, DAO activity was detectable in VTA, mildly in ML, RNu, and CP, and was absent, in clear contrast with humans, in SN as well as in SC and periaqueductal gray (PAG) (Figure 2K).

#### Pons

In humans, the basilar portion of the pons is well developed and includes tracts that conduct signals from the cerebrum down to the cerebellum and medulla, as well as tracts that carry sensory signals up into the thalamus. DAO activity was detected throughout the basilar part, with intensities descending in the following sequence: pontine nucleus (PNu), CP, and pontocerebellar fibers (PCfi). In the tegmentum of the human pons, DAO activity was observed strongly in VTT and anterolateral system (AnLS), mildly in RF, and marginally in ML (Figure 2L). On the other hand, in mice, RF occupies most of the pons. DAO activity was widely seen in RF, ML, and dorsal raphe nucleus (DRNu), whereas it was mild in lateral lemniscus (LL) and almost absent in MLF and CP in mice (Figure 2M).

#### Medulla oblongata

The medulla oblongata is structurally a site of transition between the midbrain and spinal cord, with the white matter on the outside and the gray matter on the inside. It not only relays motor and sensory fibers but also contains the nuclei of several cranial nerves. In the white matter of human medulla, DAO activity was highly observed in AnLS, anterior spinocerebellar tract (ASCT), pyramid (Py), and rubrospinal tract (RuST), while it was marginal in ML, tectospinal tract (TT), MLF, spinal trigeminal tract (STT), and restiform body (RB) (Figure 2N). In contrast, DAO activity was a trace in mouse Py (Figure 2O). In the gray matter of human medulla, olivary nucleus (OlNu), solitary nucleus (Sol), and spinal trigeminal nucleus (STNu) had moderate DAO activity; and hypoglossal nucleus (HyNu), medial vestibular nucleus (MVNu), inferior vestibular nucleus (IVNu), ambiguus nucleus (AmNu), and aucuate nucleus (ArNu) showed relatively weak activity (Figure 2N). The distribution of DAO in HyNu, STNu, Sol, AmNu, and inferior olive (IO) in mice appeared to be similar to that in humans (Figure 2O). DAO activity was undetectable in cuneate nucleus (Cu) and gracile nucleus (Gr) in mice (Figure 2O). RF, which is classified as neither white or gray matter, comprises a large portion of the medulla, and was rich in DAO activity in both humans and mice (Figures 2N,O).

#### Spinal cord

In spinal cord, DAO activity was seen in both white and gray matters. In humans, the activity in the white matter was stronger than that in the gray matter, while the activity in the gray matter was dominant in mice. Among descending pathways in the white matter of humans, we detected potent DAO activity in pyramidal tracts that consist of lateral corticospinal tract (LCST) and anterior corticospinal tract (ACST); moderate in extrapyramidal tracts, such as RuST, medullary reticulospinal tract (MReST), and pontine reticulospinal tract (PReST); and slight in vestibulospinal tract (VST) (Figure 2P). On the other hand, ascending pathways of humans showed DAO activity in the following sequence: AnLS, ASCT, posterior spinocerebellar tract (PSCT), gracile fasciculus (GrF), and cuneate fasciculus (CuF) (Figure 2P). In mice, neither ascending nor descending pathways are fully understood. DAO activity was strongly observed in ventral funiculus (VF), whereas it was detected weakly in dorsal corticospinal tract (DCS) and lateral funiculus (LF), and was almost absent in RuST, GrF, and CuF in mice (Figure 2Q). In the gray matter of humans and mice, the DAO activity in the ventral horn (VH) was relatively lower than that in the dorsal horn (DH) (Figures 2P,Q).

### Cellular distribution of DAO activity in human corticospinal tract

**Figure 3:**
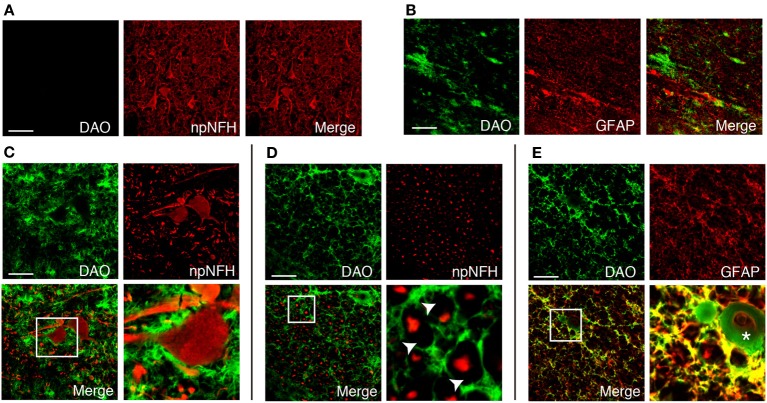
**Cellular distribution of DAO enzymatic activity in human corticospinal tract**. DAO enzyme histochemistry in the gray **(A)** or white **(B)** matter of PMC, thoracic spinal VH **(C)**, or LCST **(D,E)** in humans was double stained with npNFH, a marker for motor neurons, or GFAP, an astrocytic marker. **(C–E)** The lower right panel is higher magnification of a white square in the lower left. The arrowheads point to blanks indicating the myelin sheath. The asterisk shows blood vessel. Scale bars, 200 μm.

We found DAO activity along with motor axons throughout the corticospinal tract in humans (Figures 2B,F,J,L,N,P). In the PMC, DAO activity was absent in or around motor neurons, stained with npNFH, of the gray matter (Figure 3A), whereas the activity in the white matter was detected in GFAP-positive astrocytes in part (Figure 3B). In a similar fashion, in the spinal cord, DAO activity was not detectable in the cell bodies of npNFH-positive neurons of Lamina IX in the gray matter (Figure 3C). DAO activity in the spinal LCST existed around motor axons and myelin sheath (Figure 3D) and colocalized with GFAP-positive astrocytes (Figure 3E).

**Figure 4:**
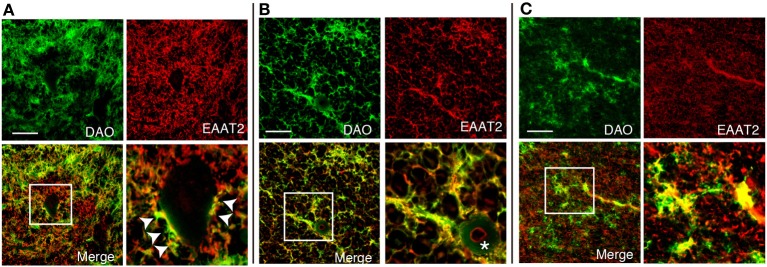
**Colocalization of DAO enzymatic activity and EAAT2**. DAO enzyme histochemistry was performed after immunohistochemistry of EAAT2 in human thoracic spinal VH **(A)**, spinal LCST **(B)**, and white matter of PMC **(C)**. The lower right image is an enlargement of the white square in the lower left image. Arrowheads show dots on the surface of a motor neuron with both DAO and EAAT2. The asterisk indicates blood vessel. Scale bars, 200 μm.

DAO is thought to regulate NMDA glutamate receptors through degrading D-serine. To find out whether DAO can contribute locationally to the D-serine level in glutamatergic synapses, DAO was co-stained with an astrocytic glutamate transporter EAAT2, which comprises 95% of the total glutamate uptake in the CNS (Tanaka et al., 1997; Williams et al., 2005). DAO-activity staining overlapped about half of the EAAT2-positive dots on the morphologically identified motor neurons in Lamina IX of the human spinal VH (Figure 4A). Not located merely on the surface of the neurons, DAO activity was merged with EAAT2-positive astrocytes for the most part in the spinal VH (Figure 4A), the spinal LCST (Figure 4B), and the white matter of PMC (Figure 4C). These colocalizations suggested the inseparable relationship between the regulations of glutamate and D-serine in controlling the activity of NMDA glutamate receptors.

## Discussion

We have shown the distribution of DAO based on its enzymatic activity in human and mouse CNS. In the forebrain, DAO activity is distributed to the cerebral white matter and internal capsule along with the ascending and descending nerve fibers in humans, while it is undetectable in mice. DAO activity is detected in the midbrain, hindbrain, and spinal cord in both humans and mice. The activity is prominent along the corticospinal and rubrospinal tracts, anterolateral and nigrostriatal systems, and ponto-/olivo-cerebellar fibers in humans. In mice, the corticospinal and rubrospinal tracts and nigrostriatal system show minimal DAO activity, whereas strong activity is detectable in the reticulospinal tract and ponto-/olivo-cerebellar fibers (Table 1). In the human corticospinal tract, DAO-activity staining colocalizes mostly with EAAT2- and partly with GFAP-positive cells.

**Table 1:** **DAO activity in major neural pathways**.

	**Human**	**Mouse**
**DESCENDING PATHWAYS**		
Corticospinal tracts	++	±
Reticulospinal tracts	+	++
Rubrospinal tracts	++	±
Vestibulospinal tracts	±	−
Tectospinal tracts	±	+
**ASCENDING PATHWAYS**		
Posterior column-ML	±	±
Anterolateral system	++	NS
Trigeminal pathways	±	±
Solitary pathways	+	+
Spinocerebellar tracts	+	NS
Ponto-/olivo-cerebellar fibers	++	++
Nigrostriatal system	++	−

*Enzyme activity: −, absent; ±, weak; +, moderate; ++, strong; NS, not specified*.

There are complexities and controversies regarding the localization of DAO in rodent brain, in terms of both region and cell type, due to differences between activity and expression. The activity-based staining, using DAO enzyme histochemistry, consistently shows that rodent DAO is a glial enzyme exclusively found in hindbrain and spinal cord (Arnold et al., 1979; Horiike et al., 1994; Sasabe et al., 2012). On the other hand, DAO immunoreactivity is also detectable in frontal cortex and hippocampus although the cerebellum and brainstem are generally more immunoreactive than the forebrain regions (Moreno et al., 1999). A possible explanation for the different results obtained using different methodologies is that DAO protein can be detected by immunohistochemistry even if it is not active. Such a difference exists also in humans. Human DAO activity can be detected only in the white matter in the forebrain region (Figures 2A–D), whereas immunoreactivity is observable in both cerebral gray and white matters (Verrall et al., 2007). Although DAO immunoreactivity is detectable in both neurons and astrocytes in the cerebral gray matter (Verrall et al., 2007; Sacchi et al., 2008), DAO is inactive in all cell types in the region (Figure 2A). Active DAO can be seen in non-neuronal cells in the white matter throughout the corticospinal tract and in the spinal gray matter (Figures 3, 4). Some non-neuronal cells with DAO activity are GFAP-negative, but most of them are EAAT2-positive (Figures 3, 4). Although GFAP in the CNS is exclusively expressed by astrocytes, up to 85% of astrocytic processes do not contain GFAP (Ludwin et al., 1976; Bushong et al., 2002). Also, susceptibility to degradation may affect the antigenicity of GFAP (DeArmond et al., 1983). Not only duration after the death, but also weak fixation of tissue for DAO-enzyme histochemistry could interfere the stability of GFAP in the process of histological analysis of human tissue. To keep DAO activity for enzyme histochemistry, we minimized fixation of tissue. But, at the same time, the weak fixation is a risk of protein degradation and loss of structural stability may result in poor labeling of GFAP by immunohistochemistry. Our results in Figures 3, 4 might just indicate that EAAT2 has better antigenicity than GFAP in the weakly fixed tissue. In either case, considering that astrocytes express high levels of EAAT2 (Lee and Pow, 2010), the DAO-positive non-neuronal cells are mostly astrocytes in the human corticospinal tract and spinal gray matter. Since the D-serine level has a clear inverse relation to DAO activity, astrocytic DAO is deemed to have a certain role in D-serine regulation in the human motor pathway.

What is the physiological significance of astrocytic DAO? It remains unclear how D-serine is regulated in DAO-rich CNS regions. Knockout studies show that D-serine is produced by its synthetic enzyme SR primarily in neurons (Miya et al., 2008; Balu et al., 2014), but it is controversial which cell types play roles in D-serine storage, release into extracellular space, and removal from it. In his seminal reviews, Wolosker explains the kinetics of serine enantiomers, called a “serine-shuttle” model, in the cerebral gray matter where DAO is basically inactive (Wolosker, 2011; Wolosker and Radzishevsky, 2013): D-serine is converted in neurons from L-serine provided by astrocytes; is released predominantly by neurons; is uptaken and stored in vesicles in astrocytes (Martineau et al., 2013); and is degraded by SR through its α, β-elimination activity in neurons. Because mice lacking DAO activity show a striking increase in D-serine in DAO-rich regions (Hashimoto et al., 1993; Morikawa et al., 2001; Miyoshi et al., 2009), not only SR but also DAO seems to be essential for D-serine regulation in these regions. Given that D-serine is synthesized in neurons also in DAO-rich regions, such as cerebellar or spinal gray matter, D-serine should be transported actively to astrocytes and degraded by astrocytic DAO in the gray matter. Thus, DAO may influence physiological NMDA receptor functions by modulating D-serine availability in the synapse. Furthermore, apart from NMDA receptor modulation, DAO may also influence δ2 glutamate receptor function, which D-serine binds to as an endogenous ligand, in cerebellar gray matter (Kakegawa et al., 2011).

On the other hand, the role of astrocytic DAO in the white matter is enigmatic. Astrocytes in the white matter, called fibrous astrocytes, have small cell bodies, and their processes align with myelinated fibers, facilitating myelination in the CNS. Since glutamate overload causes excitotoxicity via ionotropic glutamate receptors, such as NMDA receptors, not only on neuronal cell bodies or axons but also on oligodendrocytes (Oka et al., 1993; Borges et al., 1994; Karadottir et al., 2005), effective clearance of glutamate and D-serine might be required also in the white matter. In support of this view, the glutamate concentration in the white matter is only half of what is found in the gray matter *in vivo* (Hassel et al., 2003); the level of glutamate transporters is highest in astrocytes isolated from the white matter, and capacity for metabolism of glutamate to glutamine is higher in white matter astrocytes than in gray matter astrocytes *in vitro* (Goursaud et al., 2009). Because D-serine but not glycine is the dominant coagonist for NMDA receptor-mediated neurotoxicity (Shleper et al., 2005) and glycine but not D-serine is the major coagonist of “extrasynaptic” NMDA receptors (Papouin et al., 2012), degradation of D-serine by astrocytic DAO in the white matter might be necessary to maintaining healthy axons. Furthermore, D-serine triggers excitatory responses in CNS myelin through NR1/NR3 NMDA receptor (Pina-Crespo et al., 2010), suggesting that regulation of D-serine by white matter astrocytes may also affect physiology of oligodendrocytes. Considering that the distribution of human DAO activity is more widespread and prominent in the white than in the gray matter (Figure 2), in future studies, the physiological roles of DAO should be clarified in terms of modulation of extrasynaptic NMDA receptors on neurons and myelination or nerve conduction by oligodendrocytes.

Human DAO distribution in cerebral subcortical white matter and internal capsule (Figures 2A–C) implies widespread distribution along with fibers that connect between cerebral neocortex and lower brain. In contrast, in mice, such connection is much less developed and DAO activity is confined to the lower brain, suggesting that D-serine regulation through DAO is related to modulate neural network in the primitive part of brain. Since cerebral cortex has highly evolved to control the lower brain in humans, sophisticated connections are required between the two areas of brain. Considering that one of such connections, the corticospinal tract, originates not only from motor cortex but also from sensory and prefrontal cortices, distribution of DAO activity found in human subcortical white matters of these regions of cortices may imply that DAO-positive cells may have penetrated into cerebral cortex along with the evolution of human brain.

The neural pathways that show potent DAO activity imply a strong physiological association of D-serine regulation in the motor system, which consists of the pyramidal and extrapyramidal systems. The pyramidal system is also called the corticospinal tract, while the extrapyramidal system includes several neural pathways, such as the rubrospinal, reticulospinal, vestibulospinal, and tectospinal tracts. The extrapyramidal system is modulated by various parts of the CNS, including the nigrostriatal system, cerebellum, and cerebral sensory cortex. DAO activity is detectable in most of the pyramidal and extrapyramidal systems in humans, whereas it is observed mainly in the reticulospinal tract and cerebellar fibers in mice (Figure 2 and Table 1). Although in humans, the pyramidal system that connects cerebral motor cortex to spinal motor neurons is especially well developed (Lawrence and Kuypers, 1968; Lemon, 2008), there are no direct connections in rodents. Considering that in rodents, the reticulospinal tract primarily relays cortical input to spinal motor neurons (Yang and Lemon, 2003; Alstermark et al., 2004), such distributional differences of DAO between humans and mice might result from an evolutionary shift in the region responsible for voluntary movement. These differences may explain the paradox that DAO is inactivated in the reticulospinal but not the pyramidal tract in a mouse model of ALS (Sasabe et al., 2012) although in human, ALS primarily involves pathology of the pyramidal tract.

Strong DAO activity is detectable in human nigrostriatal system too, suggesting that DAO may affect not only glutamatergic but also dopaminergic neurons. Since D-DOPA is unidirectionally converted to L-DOPA mediated by DAO (Wu et al., 2006), the substrate of DAO in the nigrostriatal system might not be D-serine but DOPA, a precursor of dopamine, norepinephrine, and epinephrine. Given that DAO is overactivated in schizophrenia (Verrall et al., 2010), D-serine and dopamine being modulated by DAO may constitute a new aspect of a model of aberrant glutamatergic and dopaminergic neurons in schizophrenia.

Thus, we surveyed the regional distribution of human DAO activity in comparison to mouse DAO, which has led to a novel understanding of physiological and pathological roles of DAO in the human CNS. The cellular distribution and its specific role in each neural pathway remain largely unclear and warrant further examinations.

## Author contributions

Jumpei Sasabe and Sadakazu Aiso designed the experiments. Jumpei Sasabe, Masataka Suzuki, and Nobuaki Imanishi performed experiments. Jumpei Sasabe, Nobuaki Imanishi, and Sadakazu Aiso analyzed data. Jumpei Sasabe prepared the manuscript. All authors contributed to editing and approved the manuscript.

### Conflict of interest statement

The authors declare that the research was conducted in the absence of any commercial or financial relationships that could be construed as a potential conflict of interest.
