# Supplementary material for: Activity of D-amino acid oxidase is widespread in the human central nervous system
Source: Front Synaptic Neurosci. 2014 Jun 10;6:14. doi: 10.3389/fnsyn.2014.00014 (PMC4050652; doi:10.3389/fnsyn.2014.00014)
Supplement: Supplementary file 1 [file DataSheet1.PDF]

Supplementary Figure 1

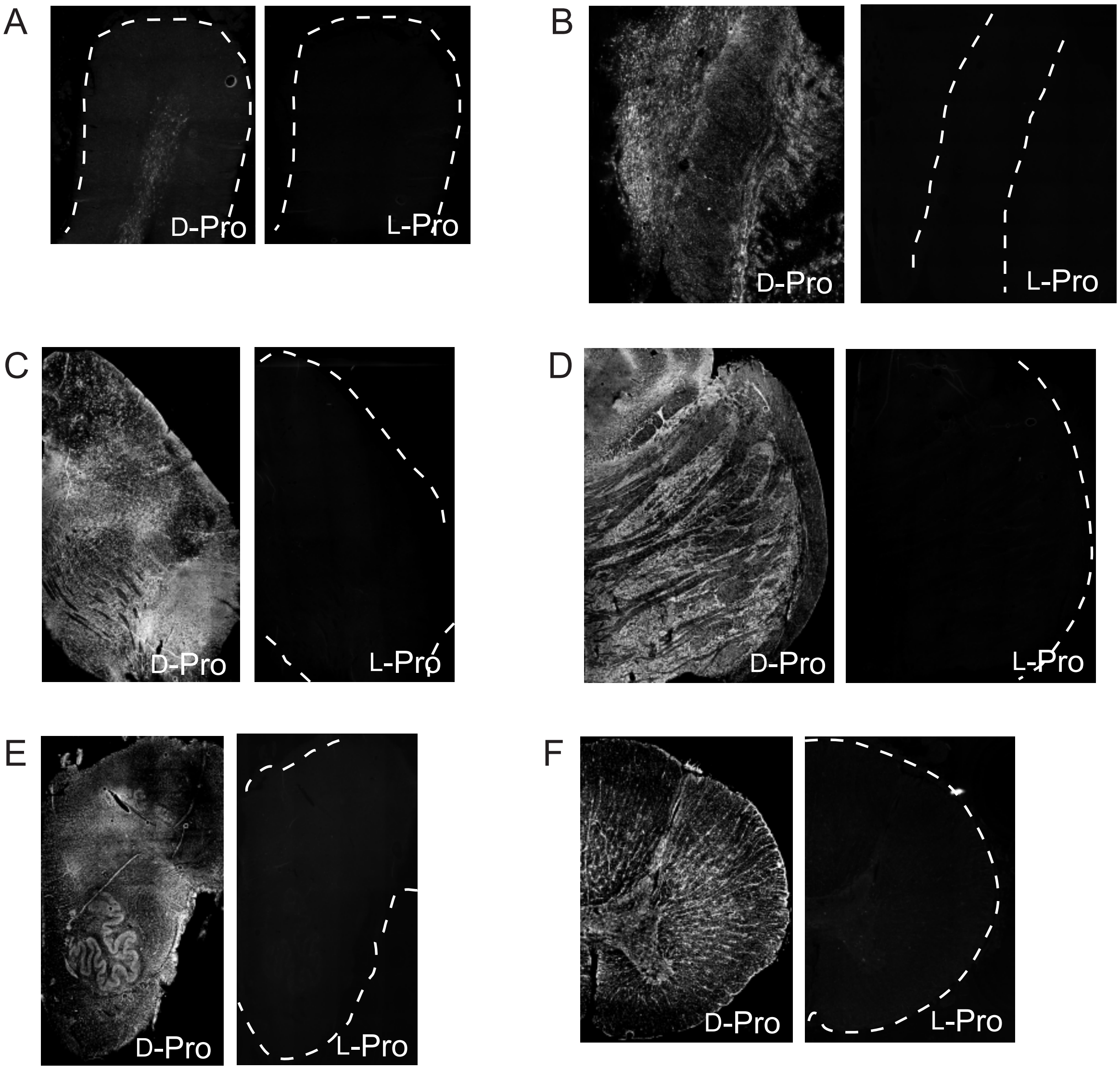

Supplementary Figure 1. Specificity of DAO enzyme histochemistry in human slices
(A-F) DAO enzyme histochemistry was performed using D- or L-proline as a substrate in human CNS tissue slices. Shown panels are (A) cerebral cortex, (B) posterior limb of internal capsule, (C) mesencephalon, (D) basilar part of pons, (E) medulla oblongata, and (F) thoracic spinal cord. Dotted lines indicate the shapes of tissue slices.
